# Supplementary material for: The characteristics of current natural foci of hemorrhagic fever with renal syndrome in Shandong Province, China, 2012-2015
Source: PLoS Negl Trop Dis. 2019 May 20;13(5):e0007148. doi: 10.1371/journal.pntd.0007148 (PMC6544330; doi:10.1371/journal.pntd.0007148)
Supplement: S1 File — (DOCX) [file pntd.0007148.s002.docx]

Some strains haven't obtained accession numbers because their L segments were not uploaded to GenBank. The partial L segment sequence of the strains without the accession number in this study are as follows:

>30F YY 2012

TATAATGTATGTCAGTGCTGATGCTACTAAATGGTCACCTGGAGACAATTCTGCAAAGTTCCGTAGGTTTACATCTATGTTACATAATGGTCTTCCCAATAATAAGTTGAAGAATTGCGTAATTGATGCACTTAAACAAGTTTACAAGACAGATTTCTTTATGTCAAGAAAATTACGAAATTATATTGATAGCATGGAAAGCCTGGACCCGCACATAAAGCAATTCTTAGATTTTTTTCCTGATGGGCATCATGGGGAAGTGAAAGGAAACTGGCTACAGGGCAATTTAAATAAATGCTCATCACTTTTCGGTGTCGCAATGTCATTGCTATTCAAACAAGTTTGGAACTGTCTATTCCCTGAGCTTGATTGCTTCTTTGAATTTGCCCACCACTCAGACGACGCAATA

>YY32 YY 2013

TTATGTATGTTTGTGCTGATGCTACGAAGTGGTCACCTGGAGACAATTCTGCAAAGTTCCGTAGGTTTACATCTATGTTACATAATGGTCTTCCCAATAATAAGTTGAAGAATTGTGTAATTGATGCACTTAAACAAGTTTACAGGACGGATTTCTTTATGTCAAGAAAATTACGAAATTATATTGATAGCATGGAAAGCCTGGACCCGCACATAAAGCAATTCTTAGATTTTTTTCCTGATGGGCATCATGGGGAAGTGAAAGGAAACTGGCTGCAGGGCAATTTAAACAAATGCTCATCACTTTTCGGTGTCGCAATGTCATTGCTTTTCAAGCAAGTTTGGAACTGTCTATTCCCTGAGCTTGATTGCTTCTTTGAATTTGCCCACCACTAGACGACGCAAGT

>SF013 LZ 2013

GTGTGTTGTACGTCAGTGCTGATGCTACTAAATGGTCACCTGGAGACAATTCTGCAAAGTTCCGTAGGTTTACATCTATGTTACATAATGGTCTTCCCAATAATAAGTTGAAGAATTGCGTAATTGATGCACTTAAACAAGTTTACAAGACAGATTTCTTTATGTCAAGAAAATTACGAAATTATATTGATAGCATGGAAAGCCTGGACCCGCACATAAAGCAATTCTTAGATTTTTTTTCCTGATGGGCATCATGGGGAAGTGAAAGGAAACTGGCTACAGGGCAATTTAAATAAATGCTCATCACTTTTCGGTGTCGCAATGTCATTGCTATTCAAACAAGTTTGGAACTGTCTATTCCCTGAGCTTGATTGCTTCTTTGAATTTGCCCACCACTCTGACGACGCCAA

>JN35 HD 2013

TAATGTATGTCAGTGCTGATGCTACAAAATGGTCACCTGGAGACAATTCTGCAAAGTTCCGTAGGTTTACATCTATGTTACATAATGGTCTTCCCAATAATAAGTTGAAGAATTGTGTAATTGATGCACTTAAACAAGTTTACAAGACAGATTTCTTTATGTCAAGAAAATTACGAAATTATATTGATAGCATGGAAAGCCTGGACCCGCACATAAAGCAATTCTTAGATTTTTTTCCTGATGGGCATCATGGGGAAGTGAAAGGAAACTGGCTACAGGGCAATTTAAACAAATGCTCATCACTTTTCGGTGTCGCAATGTCATTGCTATTCAAGCAGGTTTGGAACTGTCTATTCCCTGAGCTTGATTGCTTCTTTGAATTTGCCCACCACTCAGACGACGCA

>G01 GR 2013

CAGTGCTGATGCAACCAAATGGTCTCCAGGTGACAATTCAGCAAAATTCAGGCGATTCACTGCTGCTCTTCATAATGGATTGCCTGATGACAGGTTAAAAAATTGTGTTATTGATGCCTTGCGCCATGTATACAAGACTGACTTTTATATGTCTAGAAAACTTAGGCACTATATTGATTCTATGGATACTTATGAACCTCATGTCAGGGACTTCTTGAATTTCTTTCCAGATGGGCATCATGGAGAGGTACGAGGCAATTGGTTGCAGGGTAACTTGAACAAGTGCTCATCATTGTTTGGTGTGGCAATGTCTCTATTATTCAAAGAAATCTGGACGAGGTTATTTCCAGAATTAGACTGCTTTTTTGAATTTGCCCACCACC

>JX90L JX 2013

TATGTATGTCAGTGCTGATGCAACCAAATGGTCTCCAGGTGACAATTCAGCAAAATTCAGGCGATTCACTGCTGCTCTTCATAATGGATTGCCTGATGACAGGTTAAAAAACTGTGTTATTGATGCCTTGCGCCATGTATACAAGACTGATTTTTATATGTCTAGAAAACTTAGGCACTATATTGATTCTATGGATACTTATGAACCTCATGTCAGGGACTTCTTGAATTTCT

TTCCAGATGGGCATCATGGAGAGGTACGAGGCAATTGGTTGCAGGGTAACTTGAACAAGTGCTCATCATTGTTTGGTGTGGCAATGTCTCTATTATTCAAAGAAATCTGGACGAGGTTATTTCCAGAATTAGACTGCTTTTTTGAATTTGCCCACCACTAGACGACGCA

>1584101 HD 2015

TTCTTGTACGTCAGTGCTGATGCTACTAAATGGTCACCTGGAGACAATTCTGCAAAGTTCCGTAGGTTTACATCTATGTTACATAATGGTCTTCCCAATAATAAGTTGAAGAATTGTGTAATTGATGCACTTAAACAAGTTTACAAGACAGATTTCTTTATGTCAAGAAAATTACGAAATTATATTGATAGCATGGAAAGCCTGGACCCGCACATAAAGCAATTCCTAGATTTTTTTCCTGATGGTCATCATGGGGAAGTGAAAGGAAACTGGCTACAGGGCAATTTAAACAAATGCTCATCACTTTTCGGTGTCGCAATGTCATTGCTGTTCAAGCAGGTTTGGAACTGTTTATTCCCTGAGCTTGATTGTTTCTTTGAATTTGCTCATCATTCAGATGATGCTTTATTTATTTATGGATATTTAGAACCTGTAGATGACGGGCAGATTGGTTAA

>2015B9 QZ 2015

TTTTGTATGTTCAGTGCTGATGCCACAAAATGGTCACCTGGAGATAATTCTGCAAAATTTAGAAGGTTCACTCAAGCAATTTATGATGGTCTTCGAGATGATAAATTAAAAAATTGTGTTGTTGATGCATTAAGAAATATTTATGAAACAGAATTTTTTATGTCAAGGAAACTTCACAGGTATATAGACAATATGGGAGAACTGTCAGAAGATGTTTTAGACTTTCTCTCATTCTTTCCAAATAAAGTATCAGCAATGATAAAGGGTAATTGGCTTCAAGGAAACTTGAATAAGTGTTCTTCCTTATTTGGGGCTGCTGTTTCCCTTTTATTCAAGAGAGTCTGGTCAAAGCTTTTCCCGGAGCTAGAGTGTTTCTTTGAGTTTGCCCACCACTCTGATGATGCTTTATTTATTTATGGCTATCTAGAACCTGTTGATGACGGAACAGAATGGTTA

>AQ150218 AQ 2015

TTGTATGTTTGTGCTGATGCCACAAAATGGTCACCTGGAGATAATTCTGCAAAATTTAGAAGGTTCACTCAAGCAATTTATGATGGTCTTCGAGATGATAAATTAAAAAATTGTGTTGTTGATGCATTAAGAAATATTTATGAAACAGAATTTTTTATGTCAAGGAAACTTCACAGGTATATAGACAATATGGGAGAACTGTCAGAAGATGTTTTAGACTTTCTCTCATTCTTTCCAAATAAAGTATCAGCAATGATAAAGGGTAATTGGCTTCAAGGAAACTTGAATAAGTGTTCTTCCTTATTTGGGGCTGCTGTTTCCCTTTTATTCAAGAGAGTCTGGTCAAAGCTTTTCCCGGAGCTAGAGTGTTTCTTTGAGTTTGCCCACCACTCTGATGATGCTTTATTTATTTATGGCTATCTAGAACCTGTTGATGACGGAACAGACTGGTTA
