# Supplementary material for: The characteristics of current natural foci of hemorrhagic fever with renal syndrome in Shandong Province, China, 2012-2015
Source: PLoS Negl Trop Dis. 2019 May 20;13(5):e0007148. doi: 10.1371/journal.pntd.0007148 (PMC6544330; doi:10.1371/journal.pntd.0007148)
Supplement: S1 Table — (DOCX) [file pntd.0007148.s003.docx]

**S1** The accession number of strains used in this study.

| Label | Accession number |
| --- | --- |
| 16PG YY 2012 | KY639713 |
| L27 ZY 2013 | KY639788 |
| N14 HD 2013 | KY639785 |
| ZBLZ19 LZ 2014 | KY639751 |
| HD158453 HD 2014 | KY639779 |
| JX20141170 JX 2014 | KY639724 |
| YY140505 YY 2014 | KY639719 |
| ZY70 ZY 2014 | KY639759 |
| QZ2014D11 QZ 2014 | KY639773 |
| JX20141175 JX 2014 | KY639786 |
| ZY55 ZY 2014 | KY639787 |
| JNDX76 HD 2014 | KY639798 |
| 2015D9 QZ 2015 | KY639744 |
| 158453 HD 2015 | KY639779 |
| ZC12101 ZC 2015 | KY639780 |
| YY56 YY 2015 | KY639768 |
| 1584325 HD 2015 | KY639783 |
| 158302 PD 2015 | KY639782 |
| AQ150411 AQ 2015 | KY639752 |
| 158324 PD 2015 | KY639791 |
| GU329993 YN509 Yunnan | GU329993 |
| EU837272 CGRn2616 Guizhou | EU837272 |
| KP645196 Fj372 Fujian | KP645196 |
| KC576802 DPRK11-03 North Korea | KC576802 |
| KP900346 Rn-07-2011 Jiangxi | KP900346 |
| JQ083393 HV004 Hubei | JQ083393 |
| KP896315 JS9 Jiangsu | KP896315 |
| FJ170807 Fusong-Mf-682 Jilin | FJ170807 |
| FJ170810 Shenyang-Mf-136 Liaoning | FJ170810 |
| HM748805 HuBJ20 Bejing | HM748805 |
| KX687241 South Korea | KX687241 |
| AF336826 84FLi Xian | AF336826 |
| AF285266 Z37 Zhengjiang | AF285266 |
| DQ989237 Nc167 DBSV Anhui | DQ989237 |
| KF177177.1 DOBV RAV71 Serbia | KF177177 |
| AB677488.1 Khekhtsir37L/2002 Russia Khabarovsk | AB677488 |
| FJ170811.1 Yuanjiang-Mf-13 Hunan | FJ170811 |
| KU821030.1 JAO 449-44-15 Russia: Jewish Autonomous Oblast | KU821030 |
