# Supplementary material for: The characteristics of current natural foci of hemorrhagic fever with renal syndrome in Shandong Province, China, 2012-2015
Source: PLoS Negl Trop Dis. 2019 May 20;13(5):e0007148. doi: 10.1371/journal.pntd.0007148 (PMC6544330; doi:10.1371/journal.pntd.0007148)
Supplement: S2 Table — (DOCX) [file pntd.0007148.s004.docx]

**S2** The relationship between different partitions in Shandong Province in this study.

| Geographic  location | Regions | Surveillance sites |
| --- | --- | --- |
| southwestern Shandong Province | c | Jiaxiang |
| central Shandong Province | b | Guangrao, Linzi, Qingzhou, Yiyuan, Zichuan |
| southeastern Shandong Province | a | Anqiu, Huangdao, Jiaozhou, Jimo, Junan, Laixi, Pingdu, Zhaoyuan |
